# Medicinal, nutritional, and nutraceutical potential of *Sparassis crispa s. lat*.: a review

**DOI:** 10.1186/s43008-022-00095-1

**Published:** 2022-05-06

**Authors:** Neha Sharma, Ashwani Tapwal, Rachna Verma, Dinesh Kumar, Eugenie Nepovimova, Kamil Kuca

**Affiliations:** 1 Himalayan Forest Research Institute, Conifer Campus, Panthaghati, Shimla, 171013 India; 2 School of Biological and Environmental Sciences, Shoolini University of Biotechnology and Management Sciences , Solan, Himachal Pradesh 173229 India; 3 School of Bioengineering and Food Technology, Shoolini University of Biotechnology and Management Sciences , Solan, Himachal Pradesh 173229 India; 4 Department of Chemistry, Faculty of Science, University of Hradec Kralove, 50003 Hradec Kralove, Czech Republic

**Keywords:** Anti-cancer, Artificial cultivation, β-glucan, Cauliflower mushroom, Immunomodulatory, Sparassol

## Abstract

*Sparassis crispa *is an edible mushroom exhibiting a wide range of medicinal properties. It is recognized for therapeutic value because of the high β-glucan content in the basidiomes. The broad range of its reported curative effects include anti-tumour, anti-cancer, immune-enhancing, hematopoietic, anti-angiogenic, anti-inflammatory, anti-diabetic, wound-healing, antioxidant, anti-coagulant, and anti-hypertensive properties. However, most of the studies are conducted on immunomodulatory and anticancer activities. Besides this, it also exhibits anti-microbial properties due to the presence of sparassol. Technology is now available for the cultivation of *S. crispa* on coniferous sawdust. This review is an attempt to focus on its distribution, taxonomy, chemical composition, medicinal properties, potential applications, and artificial cultivation.

## INTRODUCTION

Medicinal mushrooms are macroscopic fungi used in the mitigation, prevention, and curing of diseases. Mushrooms are a rich source of proteins, essential amino acids, vitamins, and minerals; and have a wide spectrum of uses as food and medicine. They can be used in the form of extracts or directly included in the diet. Mushrooms have been used in medicine since ancient times and this potential has led to them gaining popularity in current times. Medicinal properties can be attributed to the low-fat content, with a high proportion of unsaturated fatty acids along with high fibre content, triterpenes, phenolic compounds, sterols, eritadenine, and chitosan. Glucans (β-d-glucans), triterpenoids, and ergosterol are key active compounds. Mushrooms have also gained importance in medicine as they contain vital components which provide nutrition and health benefits for humans (Ogidi et al. 2020).

*Sparasis crispa,* commonly known as cauliflower mushroom after the shape of the above ground basidiomes which resembles a cauliflower. An edible mushroom with a variety of medicinal properties, it is widely distributed all over the north temperate zone and grows as a saprophyte or parasite on the stumps of conifers and some hardwoods, especially oaks causing a brown rot, and the basidiomes are generally seen on or near the base of a tree trunk. The species is well known for its medicinal significance arising from a variety of pharmacologically active substances and incorporated into health supplements. It is traditionally used in Chinese medicine. Medicinal properties are primarily due to a high β-glucan (sparan) content which can constitute more than 40% of the dry weight of the mushroom (Ohno et al. 2000).

It is reported as exhibiting antitumor, anti-angiogenic, immunity enhancing, wound-healing, hematopoietic, antihypertensive, and antioxidant activities (Harada et al. 2002a; Harada et al. 2006; Tada et al. 2007; Yamamoto et al. 2009; Kwon et al. 2009). Besides this, it also contains sparassol (methyl-2-hydroxy-4-methoxy-6-methylbenzoate), which has antibacterial and antifungal properties. *Sparassis crispa* has a distinctive aromatic scent that can be attributed to 3-octanone, DL-3-octanol, and 1-octen-3-ol (Shin et al. 2007). It also has the potential to be used in the food industry as it contains hydrocolloid (Hao et al. 2018; Vaka et al. 2020). Artificial cultivation of *S. crispa* is practiced in many parts of the world.

## TAXONOMY AND MORPHOLOGY

The generic name *Sparassis* is derived from a Greek word meaning ‘to tear’, because the fronds appear as if they are torn. The genus was first described in 1821 by the Swedish mycologist Elias Fries, and includes species that have an amphigenous hymenium and give rise to flabellae from a central mass (Wang et al. 2004). The specific epithet *crispa* recalls the finely curled lobes of the basidiome, which are quite pliable and cartilaginous in texture. Index fungorum (http://www.indexfungorum.org/) lists 17 species names under this genus, but molecular phylogenies have reduced the number to ten (Zhao et al. 2013). The genus belongs to the *Sparassidaceae* (*Polyporales*, *Agaricomycetes*). It was earlier described as *Elvela ramosa* (Schaeffer, 1772) then Wulfen (1781) described it as *Clavaria crispa* and it was subsequently transferred to *Sparassis* by Fries (1821, Burdsall & Miller 1988). More recent insights into the taxonomy of genus *Sparsassis* relating taxa to geographical distribution is provided by Dai et al. (2006) and Ryoo et al*.* (2013). Early research on *S. crispa* from Asian countries appears to reflect the *S. crispa s*. *lat.* monophyletic clade, but based on morphological and molecular studies material named as *S. crispa* from Asia is different to that from Europe and North America. The predominant Asian isolate was therefore named *S. latifolia,* based on morphological characters and molecular sequence analysis of the rDNA, particularly the internal transcribed spacer (ITS) region (Ryoo et al. 2013).

In the present review, because of this issue, the name “*S. crispa”* refers to the species in the broad circumscription unless otherwise indicated.

It is important that the identification of fungal species used in experimental and other studies is authenticated using molecular methods, and that representative material be deposited in a public fungarium or culture collection so that it is available to check the identities of the fungi as well as for future research. The metadata linked with specimens in fungaria and culture collection is now becoming more publicly available as a result of large-scale digitization. Taxonomy, sampling location, collection date, and habitat/substrate information, as well as DNA sequences and biochemical properties, can all now be used to their maximum potential (Andrew et al. 2018).

### Anatomical features

*Basidiome* large, rounded and branched with leaf-like projections called flabellae; flabellae densely grouped, flat, waxy, undulating, fleshy, emerging from a branched central base, whitish to cream in color, becoming darker with age; hymenium amphigenous, surface smooth (Fig. A). *Hyphae* septate, thin-walled, monomitic, branched, hyaline, 4–6 µm wide, clamp connections present. *Basidia* club-shaped, sterigmata 4, 2–5 µm tall. *Basidiospores* smooth, thin-walled, ovoid, hyaline, with a central guttule, 6.0–7.5 × 4.0–5.0 µm, non-amyloid (Fig. B). *Spore print* white to cream (Rana 2007).

### Culture characteristics

**Fig. 1:**
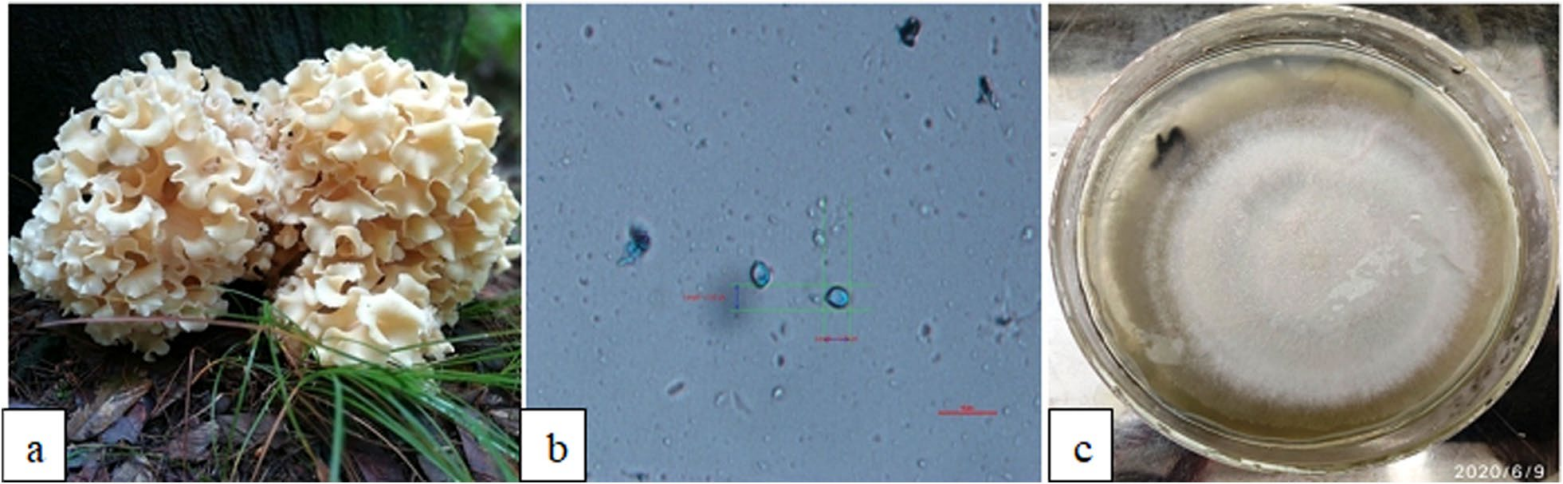
**a** *Sparassis crispa basidiomes* at the base of a conifer. **b** Spores at 100X magnification. **c** Mycelial colony of *S. crispa* after 15 d incubation at 25 °C on PDA

Slow growing, colony is circular, cottony, initially white, later on, turn pale on ventral side, form dense and sparse zones of concentric rings, margins smooth. It grows to a diameter of 75 mm on PDA medium, after 15 days of incubation at 25 °C (Fig. 1c).

## GENOME SEQUENCE AND PHYLOGENY OF *SPARASSIS CRISPA*

According to Kiyama et al. (2018), *S. crispa* has a 39.0 Mb genome, encoding 13,157 genes. Phylogenetic analysis suggests that about 94 Mya it diverged from *Postia placenta*, a resupinate brown-rot fungus. Bashir et al. (2020) reported a complete mitochondrial genome sequence of *S. crispa.* including 139,253 base pairs with 26.47% Guanine-Cytosine and 73.53% Adenine–Thymine content. It contains 47 genes which include 27 tRNA, 5 ribosomal RNA genes and 15 protein-coding genes. The phylogenetic analysis on *atp6* sequence revealed a close relationship with *S. radicata *(Dai 2006).

## ECOLOGY AND DISTRIBUTION

*Sparassis crispa* is considered a biotroph, weak parasite, and saprotroph in nature (Julich 1984). It invades the roots of conifers and some hardwood tree species causing brown heart rot. The fungus thrives in forests having fine soil and high moisture (Park et al. 2009). *Sparassis crispa* basidiomes can be spotted during July to October at or near the base of host trees. *S. crispa s. lat.* is found in the USA, Australia (Martin & Gilbert 1976), France, Germany, Russia (Dyakov et al. 2011), China, Japan, Tibet, Nepal (Devkota 2009), Belarus, Turkey (Kalyoncuet al. 2010) and India (Lakhanpal 2014). In India, it is most commonly found in Himachal Pradesh (Chauhan et al. 2014), Uttarakhand (Kumar et al. 2017), Jammu-Kashmir (Lalotra et al. 2018) and Maharashtra (Fulzele 2013).

## PATHOGENICITY AND HOST RANGE

*Sparssis crispa* does not cause major harm to trees, and infected trees can live for many years supporting newly grown mushrooms annually. It is a weak parasite and has little ability to invade the living tissues of host plants (Woodward et al. 1993). It is generally found on physiologically weakened or recently killed trees, logs, and thick fallen branches. The fungus mostly colonizes the living trees through roots and causes brown cubical rot of root and stem. The rot progresses from the root to the heartwood and gradually invades the lower part of the tree (Karadžić, 2006). The presence of wounds on the trunk and roots facilitates the entry of the pathogen (Park et al. 2009). Oh et al. (2009) surveyed the natural habitat of *S. crispa* for investigating ecological conditions. *S. crispa* was found on *Larix kaempferi* and *Pinus koraiensis* tree species growing in relatively fertile forest soils and especially slightly acidic soils. *Pinus nigrum* and *Pinus sylvestris* are especially susceptible to this fungus (Radulovic et al. 2019). Few common hosts of *S. crispa* reported by different researchers are listed in Table 1.

**Table 1:** Tree species invaded by *S. crispa*

Host tree	References
*Abies holophylla*	Ryu et al. (2009)
*Cedrus deodara*	Semwal et al. (2014)
*Ceiba pentandra*	Kamble et al. (2012)
*Larix deciduas*	Woodward et al. (1993)
*Larix kaempferi*	Ryu et al. (2009); Oh et al. (2009)
*Melia azadarachta*	Nasim et al. (2007)
*Picea* sp.	Wang et al. (2004)
*Pinus brutia*	Kalyoncu et al. (2010)
*Pinus densiflora*	Ryu et al. (2009); Farooq et al. (2014)
*Pinus koraiensis*	Dai et al. (2007); Ryu et al. (2009); Oh et al. (2009)
*Pinus nigra*	Martin and Gilbertson (1976)
*Pinus sylvestris*	Burdsall and Miller (1988)
*Pinus virginiana*	Martin and Gilbertson (1976)
*Pseudotsuga menziesii*	Von-Siepmann (1976)
*Quercus alba*	Martin and Gilbertson (1976)
*Quercus lamellose*	Devkota (2009)
*Quercus semecarpifolia*	Devkota (2009)
*Tectona grandis*	Aryal and Budhathoki (2013)
*Tsuga Dumosa*	Devkota (2009)

## ETHNOMYCOLOGY

*Sparassis crispa* is considered edible all over the world. It is usually consumed in a young fresh condition, but sometimes the mushrooms are dried and preserved for future use. It has been used in China, Japan, and Korea in traditional culinary practices for centuries. In India, it is reported as edible from Jaunsar, Uttarakhand (Kumar et al. 2017); Kinnaur, Himachal Pradesh (Chauhan et al. 2014), and Jammu-Kashmir (Lalotra et al. 2018; Aryal & Budhathoki 2013).

## CHEMICAL CONSTITUENTS

*Sparassis crispa* basidiomes are primarily composed of proteins, lipids, carbohydrates, and minerals. The major part is of carbohydrates, which have the highest content of β-glucan. It is estimated that more than 40% of the dried basidiomes are composed of β-glucan (Ohno 2000). More than 30 compounds have been isolated from *S. crispa,* including polyphenols, flavonoids, terpenoids (Kodani et al. 2009), vitamins, steroids (Lee et al. 2016c), alkaloids, phthalides (Yoshikawa et al. 2010), and some others (Kawagishi et al. 2007). Shin et al. (2007) analyzed the minerals, amino acids, and vitamin content of the *S. crispa* basidiomes and found that the mushroom possessed a high amount of potassium (K) and appreciable amounts of phosphorus (P) and sodium (Na). Among the amino acids, glutamine was present in the highest amount followed by asparagine. The mushroom also contains a high amount of vitamin E and vitamin C.

Indole, tryptamine, melatonin, gentisic acid, gallic acid, p-hydroxybenzoic acid, o-cumaric acid, caffeic acid, protocatechuic acid, syryngic acid, and ergosterol are all antioxidant compounds reported from *S. crispa.* Among these, p-Hydroxybenzoic acid was recorded to be the most abundant with 43.92 mg per 100 g dry weight of the basidiome (Kawagishi et al. 2007).

Kavishree et al*.* (2008) investigated the fatty acids of *S. crispa* and some other Indian edible mushrooms and, recorded a high content of essential fatty acids in this mushroom. Hayashi et al. (2008) reported two chalcones, xanthoangelol and 4-hydroxyderricin, from *S. crispa*. Yoshikawa et al. (2010) isolated phthalid compounds; ubiquinone-9, hanabiratakelide A, hanabiratakelide B, hanabiratakelide C, and two unsaturated compounds. Other than these compounds, sparalide A, methyl 2,4-dihydroxy-3-methoxy-6-methylbenzoate, and 50-deoxy-50-methylthioadenosine, were isolated from *S. crispa* by Bang et al. (2018). Chandrasekaran et al. (2011) found an alkaliphilic esterase from the basidiomes of *S. crispa* which shows high specificity towards moderate thermostability, short-chain esters, and alkaliphilic properties; it consequently has potential as a biocatalyst in the pharmaceutics, food, and chemical industries. Choi et al. (2016) isolated a fibrinolytic enzyme wulfase, and Nowacka-Jechalke et al. (2021) observed a dominant presence of carbohydrates while other components were seen in traces.

Horie et al. (2008) carried out a proteomics analysis of *S. crispa* using one and two dimensional gel electrophoresis-based complementary proteomics approaches. Using 1-DGE and 2-DGE in combination with liquid chromatography, Mass spectroscopy, and N-terminal amino acid sequencing, 115 proteins were identified from the mushroom.

## MEDICINAL PROPERTIES

Several authors have summarized the pharmacological potential of *S. crispa *viz. anti-tumour (Ohno et al. 2000, 2002; Yamamoto et al. 2007), anti-cancer (Ohno et al. 2003; Yamamoto et al. 2009; Yoshikawa et al. 2010), immune-enhancing (Hasegawa et al. 2004; Kim et al. 2010) hematopoietic (Harada et al. 2006; Harada et al. 2002a, b), anti-angiogenic (Yamamoto et al. 2009), anti-inflammatory (Yao et al. 2008; Kim et al. 2012; Wang et al. 2019; Han et al. 2019), anti-diabetic (Yamamoto & Kimura 2010), wound-healing (Kwon et al. 2009; Wang et al. 2019) antioxidant (Kim et al. 2008; Joshi & Sagar 2014; Lee et al. 2016a, b, c), anticoagulant (Choi et al. 2016) and antihypertensive (Yoshitomi et al. 2011). The bioactivities of *S. crispa* are mainly atributted to the presence of a high amount of β-glucan. "Phytochemicals” from the mushroom *S. crispa* were also observed to inhibit LPS-stimulated cytokine production in bone marrow-derived dendritic cell (Bang and Lee 2019). Nowacka-Jechalke et al. (2021) confirmed that crude polysaccharide extracts from *S. crispa* possess many biological activities including anticancer, anti-inflammatory, and antioxidant properties. The different medicinal properties associated with particular chemical constituents of *S. crispa* are summarized in Table 2

**Table 2:** Medicinal properties of different chemical constituents present in *S. crispa*

Chemical constituents	Properties	Reference
Indole, tryptamine, melatonin, gentisic acid, gallic acid, p-hydroxybenzoic acid, o-cumaric acid, caffeic acid, protocatechuic acid, syryngic acid, and ergosterol	Antioxidant activity	Kawagishi et al. (2007)
Crude polysaccharides		Nowacka-Jechalke et al. (2021)
alkaliphilic esterase	used as a biocatalyst in the pharmaceutics, food, and chemical industries	Chandrasekaran et al. (2011)
Crude polysaccharides	Anticancer activity,	Nowacka-Jechalke et al. (2021)
1,3-β-D glucan	Anticancer activity,	Ohno et al. (2000)
	used in cancer treatment	Harada et al. (2006)
polysaccharide fractions of 1,3 β-glucan	antitumor activity	Ohno et al. (2000)
the β-glucan fraction CA1	Anticancer activity	Ohno et al. (2002)
3 novel phthalides- hanabiratakelide A (1), B (2), and C (3)	Antiallergic against rhinitis	Yoshikawa et al. (2010)
Sparoside A	Antiallergic against rhinitis	Wang et al. (2019)
β-glucan	wound healing capacity	Kimura et al. (2013)
Enhances increased production of adiponectin	Antidiabetic: regulates glucose levels and fatty acid breakdown	Yamamoto and Kimura (2010),
Xanthoangelol, 2 chalcones, and 4-hydroxyderricin	Anti-bacterial activity	Hayashi et al. (2008)
Sparassol and the other two were identified as incompletely determined methyl-dihydroxy-methoxy-methylbenzoate and methyl orsellinate	Anti-fungal activity (against *Candida albicans*)	Woodward et al. (1993)
Enhances NO production	Anti-hypertensive activity	Yoshitomi et al. (2011)

### Anti-cancer activity

The important immunomodulator 6-branched 1,3-β-D glucan exists as an active compound in *S. crispa*, and exhibits anticancer activity by modulating the immune response (Ohno et al. 2000). The major side-effect reported from most of the anti-cancer chemotherapeutic drugs is neutropenia and impairment of blood-forming functions. The β-glucan is known to enhance the hematopoietic response and therefore has potential application in cancer treatment (Harada 2006).

Ohno et al. (2000) recorded antitumor activity against a solid form of sarcoma in ICR mice (a strain of albino mice originated in SWISS and selected to create a fertile mouse line**)** by polysaccharide fractions of 1,3 β-glucan extracted from *S. crispa*. According to Ohno et al. (2002), oral administration of the β-glucan fraction CA1 (extracted with cold NaOH), enhanced the anticancer activity by influencing the hematopoietic response in leukopenic mice (after cyclophosphamide induction), thereby increasing white blood cell count. *In-vitro* investigation of CY (cyclophosphamide) treated spleen cell culture recorded higher production of interleukin-6 and interferon-γ, on treatment with CA1. The enhancement in hematopoietic response can be attributed to increased cytokine production. Harada et al. (2002a, b) also reported enhancement of the hematopoietic response in CY (Cyclophosphamide) induced leukopenic mice by intraperitoneal administration of SCG (a purified beta-glucan preparation). The recovery rate of monocytes and granulocytes in the spleen, liver, peritoneal cavity, and bone marrow (BM) was higher than control. The concentration of natural killer cells and γδ T cells in the spleen, liver, and peritoneal cavity was also increased. They opined that IL-6 might be the key cytokine for the enhanced hematopoietic response by SCG because its production was more in SCG treated CY mice. On administration of SCG, splenocytes from naive DBA/1 (an inbred strain of mice susceptible to arthritis) and DBA/2 mice (another inbred strain) were reported to have reacted with it and produced interferon-*γ* (IFN-*γ*) (Harada et al. 2002b). Ohno et al. (2000) examined the cytokine-inducing capacity of SCG in healthy human volunteers and recorded increased cytokine synthesis of whole blood cell culture in dose-dependent manner. Complement system component (C5a), was released by SCG in a dose dependent manner. In a clinical trial, oral administration of powdered *S. crispa* (300 mg/day) to several cancer patients, after one course of lymphocyte transfer immunotherapy, proved beneficial. Patient assessment of 14 cases after months of follow-up period revealed that nine showed improvement in the quality of life (Ohno et al. 2003). Nameda et al. (2003) tested the effect of SCG on white blood cells collected from human volunteers and observed that SCG dose-dependently enhanced IL‐8 synthesis in both PMN (polymorphonuclear neutrophil) and PBMC (peripheral blood mononuclear cell) cultures. Therefore β-glucan can activate human leukocytes and related immune systems.

Harada et al*.* (2004) screened the cytokines induced by SCG, which includes granulocyte–macrophage colony-stimulating factor (GM-CSF), tumour necrosis factor-*α* (TNF-*α*), IFN-*γ*, and interleukin-12 (IL-12p70). Hasegawa et al. (2004) examined the immunomodulatory effect of β-glucan isolated from *S. crispa*, and reported that oral administration reduced the tumour size of sarcoma 180-bearing mice after 5 wk, as well as prolonged their survival. Oral administration of FHL (low molecular weight fraction) is also shown to induce antitumor activity by enhancing the Th1-response in tumour-bearing mice (Yamamoto et al. 2007). It also suppressed the tumour-induced angiogenesis in the dorsal air sac (DAS) system which may have contributed to the antitumor activity of FHL. β glucan activates dendritic cells via NF-κB signaling and Mitogen-Activated Protein Kinase (MAPK) pathways and thus helps in its maturation. Oral administration of the purified β-glucan extract (SBG) resulted in the suppression of tumour growth and metastasis in the lungs by inhibiting tumour-induced angiogenesis (Yamamoto et al. 2009). Kim et al. (2012) observed that β-glucan brings about phenotypic and functional maturation of dendritic cells, which are antigen-presenting cells of the immune system. Mycelia of *S. crispa* showed less tumour-suppressing activity compared with that of the basidiome. This difference in antitumor activity in between mycelia and basidiome of *S. crispa* might be attributed to the difference in structure and content of β-glucan (Kimura et al. 2013). Nowacka-Jechalke et al. (2021) carried out an in vitro study to evaluate the beneficial effects of crude polysaccharides from *S. crispa* against colon cancer. They observed that polysaccharides destroyed membrane integrity and inhibited the proliferation of human colon cancer cell lines: HT-29, LS180, and Caco-2. Therefore, it may be suggested that *S. crispa* contributes to reduction in the risk of various cancers. The stipe of *S. crispa* is also reported to exhibit immunomodulatory activity (Seo et al. 2016).

### Anti-inflammatory activity

Allergic inflammatory diseases, such as asthma, allergic rhinitis (hay fever), food allergy, and atopic dermatitis, are quite prevalent across the globe. Some researchers have demonstrated anti-inflammatory activities in *S. crispa*. Hasegawa et al. (2004) reported that oral administration of *S. crispa* lowered the IgE level and scratching index of NC/Nga mice, which was induced by dermatitis (skin inflammation).

Anti-rhinitis activity of β-glucan is reported by many workers. Allergic rhinitis is a type of inflammatory condition which affects the nose due to overreaction of the immune system to air allergens. It is characterized by elevated production of IgE and mast cell degranulation, which results in histamine release. Yao et al. (2008) observed that oral administration of *S. crispa* (ultrafine powderfrom basidiomes) to mice had shown reduced symptoms of allergic rhinitis in a dose-dependent manner. Yoshikawa et al. (2010) isolated three novel phthalides- hanabiratakelide A (1), B (2), and C (3) from the basidiomes of *S. crispa* which exhibited antiinflammatory activity. Kim et al. (2012) reported anti-inflammatory activity of water extracts of *S. crispa* (containing 39.6% β-glucan) on mast cell-mediated allergic inflammation in mice. Extracts play an inhibitory role in inflammatory reactions by regulating NO production (Sekiguchi 2005). Han et al. (2019) reported an anti-inflammatory effect of the non-aqueous fraction of an *S. crispa* methanol extract (SCF4) in lipopolysaccharide (LPS)-stimulated (RAW264.7) murine macrophage cells. Wang et al. (2019) reported the potential inhibitory effect of compounds from *S. crispa* on allergic rhinitis. Sparoside A was found to be the active anti-inflammatory compound with an IC50 value of 5.06 ± 0.60 µM.

### Anti-diabetic and wound healing activity

Yamamoto and Kimura (2010), after feeding type 2 diabetic KK-Ay mice with an *S. crispa* diet for 3–6 wk, observed a significant increase in production of adiponectin, a protein hormone involved in regulating glucose levels and fatty acid breakdown in the plasma of diabetic mice. A substantial decrease in blood glucose and insulin levels was recorded after 3 wk administration of *S. crispa*, along with somewhat decreased levels of triglycerides and total cholesterol, which otherwise are elevated in diabetes. Yoshitomi et al. (2011) observed a preventive effect of *S. crispa* against hypertension and stroke in hypertensive rats. As the fungus alleviates dysfunctioning of the cerebrovascular endothelia by promoting recovery of Akt-dependent endothelial nitric oxide (eNOS) phosphorylation, it also increases NO production in the cerebral cortex.

The wound healing process is generally delayed or impaired in diabetic patients. β-glucan increases the macrophage infiltration and collagen biosynthesis and thus enhances wound healing capacity (Kimura et al. 2013). Neutrophils, keratinocytes, fibroblasts, endothelial cells, and macrophages are all important cells in wound repair, and pattern-recognition receptors for β-glucans have been identified on these cells (Lowe et al. 2002). β-glucans mediate their effects by activating leukocytes and stimulates their phagocytic activity. Dectin-1 is the major receptor for β-glucans on leukocytes (Choi et al. 2013).

Kwon et al. (2009) tested the wound healing property of *S. crispa* on STZ (streptozotocin) induced type 1 diabetic rats and observed that oral administration of *S. crispa* accelerated wound healing*. *This effect was linked to an increase in macrophage and fibroblast migration as compared to control. Moreover, the level of type I collagen was also higher in the treated group. Yamamoto and Kimura (2013) also reported that a diet supplemented with more than 0.5% *S. crispa* (ultra-fine powderof basidiomes) improved wound healing in diabetic mice. Topical administration of purified *S. crispa* β-glucan (SBG) also improved wound healing, resulting in a wound contraction ratio of 37% after 9 d of treatment.

Sharifi-Rad et al. (2020) also observed wound healing property in *S. crispa.* Oral administration of *S. crispa* (with a β-glucan content more than 40%) at a dose of 1 mg/kg b.w. per day for 4 wk, healed the wound through macrophage and fibroblast migration, increasing collagen regeneration, and wound epithelialization in STZ-induced diabetic rats.

### Antioxidant activity

Oxidation is essential in many living organisms for energy production to fuel biological processes. However, uncontrolled production of oxygen leads to the release of free radicals which can induce many diseases, such as cancer, rheumatoid arthritis, and atherosclerosis, as well as accelerating the aging process. Antioxidants present in dietary mushrooms can act as possible protective agents to help the human body reduce oxidative damage without any interference (Dhalaria et al. 2020). The phenolic compounds of mushrooms are known to have excellent antioxidant activity. Antioxidant activity of a compound can be assessed through a number of assays which include DPPH (2, 2-diphenyl-1-picrylhydrazyl) radical scavenging activity and oxidative-inhibitory capacity. Phenolic compounds and flavonoids are the major antioxidant compounds from *S. crispa* (Joshi & Sagar 2014). p-hydroxybenzoic acid, a phenolic derivative of benzoic acid, was found to be the quantitatively predominant antioxidant compound in *S. crispa* by Sułkowska-Ziaja et al. (2015)*.* Crude polysaccharides from the mushroom are also reported as having antioxidant activity (Nowacka-Jechalke et al. 2021).

Puttaraju et al. (2006) analyzed the antioxidant activity of water and methanolic extracts of *S. crispa* basidiomes and other mushrooms for total anti-oxidative status, based on the inhibitory effect on lipid peroxidation, reducing power, and free radical scavenging activity. Moderate antioxidant activity was observed in water and methanolic extracts of *S. crispa* basidiomes. The phenolic content in *S. crispa* was observed as 5.5 mg/g (in water extract) and 1.7 mg/g (in methanolic extract). Kim et al. (2008) evaluated the antioxidant activity by DPPH scavenging activity and superoxide dismutase activity assay. Lee et al. (2016a, b, c) compared the antioxidant compounds and antioxidant activities of the *S. crispa* mycelium, based on extraction temperature (60 °C and 95 °C). That study revealed that the antioxidant activity of *S. crispa* extracted at 95 °C was better than that extracted at 60 °C, and the mycelium contained more antioxidant compounds in comparison to the pileus and stipe. Lee et al. (2016a, b, c) reported that *S. crispa* fermented with lactic acid bacteria (LAB) showed higher antioxidant activity. The activity was even higher when fermented with *Meyerozyma guilliermondii* (Park et al. 2016).

### Anti-viral activity

Wang et al. (2007) investigated the effect of the hot water extracts of *S. crispa,* and 15 other mushroom species, on human immune deficiency virus (HIV)-1 reverse transcriptase (RT) activity. The extracts, at a concentration of 1 mg/ml, exhibited 70.3% inhibition.

### Antibacterial activity

Hervey (1947) reported antibacterial activity of *S. crispa* against *Staphylococcus aureus* and *Escherichia coli*. *S. crispa* is also known to possess antimicrobial activity against many bacterial species including *Bacillus mycoides, B. subtilis, B. pumilis, Comamonas terrigena,* methicillin-resistant *Staphylococcus aureus* (MRSA)*, Micrococcus luteus,* and *Leuconostoc mesenteroides* (Dyakov et al. 2011). Hayashi et al. (2008) isolated xanthoangelol, two chalcones, and 4-hydroxyderricin from extracts of *S. crispa* exhibiting anti-MRSA activity. Lee et al. (2013a) reported antimicrobial activity against some food poisoning bacteria, viz. *B. cereus, Listeria monocytogenes, Salmonella typhimurium,,* and *Staphyllococcus aureus. *The anti-microbial activity was maintained even after heating and treating with alcalase, indicating potential as a natural preservative in the food industry. Niazi and Ijaz (2021) evaluated the antibacterial potential of the ethanolic extract of *S. crispa,* which had a maximal effect against *E. coli*, with a potential inhibition zone (19.66 ± 0.88 mm). Jang et al. (2015) observed growth inhibition of MRSA by *S. crispa*.

### Anti-fungal activity

Woodward et al. (1993) isolated three antifungal metabolites from submerged cultures of *S. crispa* in 2% malt broth’ sparassol (methyl-2-hydroxy-4-methoxy-6-methylbenzoate) was one of the compounds and the other two were identified as incompletely determined methyl-dihydroxy-methoxy-methylbenzoate, and a methyl-2,4-dihydroxy-6-methylbenzoate (methyl orsellinate). These compounds were also reported from the decayed wood of trees infected by *S. crispa*. Jang et al. (2015) observed growth inhibition of *Candida albicans* by *S. crispa*.

### Antihypertensive activity

Hypertension, high blood pressure, is the most prevalent risk factor for stroke. Therefore, as a preventative measure for controlling a stroke one must control high blood pressure. The impact of *S. crispa* is well proved against hypertension and stroke in stroke-prone spontaneously hypertensive rats (SHRSP) (Yoshitomi et al. 2011). The oral administration of 1.5% *S. crispa* to SHRSP from a young age delayed stroke and decreased blood pressure. The increased NO production acts as the main mechanism behind this, which improved the endothelial dysfunction with activation of the Akt/eNOS signaling pathway on the cerebral cortex.

### Anti-obesity activity

*Sparassis. crispa* also prevents hepatic steatosis by lowering lipid content through activation of the beta-oxidation pathway. Experimentation was based on the detection of *Lactobacillus*-fermented *S. crispa* against obesity using a zebrafish model. The L-SC (lacto-fermented *S. crispa*) or orally administered *S. crispa* was subjected to diet-induced obese zebrafish for 4 weeks which resulted in suppression of body weight (20 μg/gBW/day) significantly (P < 0.01) and ameliorated lipid accumulation in liver tissues (Matsuura et al. 2020).

## CULTIVATION

Availability of *S. crispa* is scanty in nature, therefore artificial cultivation is practiced in many countries. At present, it is commercially cultivated in Japan, Korea, China, Germany, and the USA, mainly on sawdust of *Larix* trees. It can be cultivated by bottle, bag, and log cultivation methods using different solid and liquid media. The optimum temperature for mycelial growth is 20–25 °C, the optimum pH is slightly acidic (4–7), optimum atmospheric humidity 90–95% (Huang et al. 2007; Yu et al. 2010), and substrate moisture content of 65% (Yanquan et al. 2012) is considered best for its growth.

Shim et al. (1998) were probably the first to carry out studies to obtain basic information for the artificial culture of *S. crispa*. A pH of about 4 and a temperature around 25 °C was observed to be best for mycelial growth. Hamada media showed the most compact mycelia, while colony diameter was the largest in Hoppkins media. Park et al*.* (2006) reported that steam treatment of sawdust minimizes the culturing period and increases the productivity of mushrooms. He used sawdust medium of *Larix leptolepsis, Pinus densiflora,* and *P. koraiensis*. Seo (2003) recorded an increase in thickness and growth of basidiomes by using light illumination (600–10,000 lx) in a culture medium comprised of conifer and broad-leaved tree sawdust in a ratio of 5:1 to 3:1 by weight. Lee et al*.* (2004) screened different sawdust media for suitability formycelial growth. Six different kinds of sawdust were prepared, from oak *(Quercus mongolica)*, mulberry (*Morusalba*), poplar (*Populus deltoides*), larch (*Larix kaemferi),* pine (*Pinus densiflora*), and Korean white pine (*P. koraiensis*). Amongst these, sawdust made from *Larix kaemferi* was observed as giving the highest mycelial growth. Sekiguchi (2005) cultured *S. crispa* hyphae in an agar medium and prepared seed culture by using sawdust of conifers or mixed sawdust of conifers and deciduous trees. Farooq et al*.* (2014) reported that the application of electric pulse had a significant effect on basidiomes production and that the yield increased with increasing voltage of the electric pulse. Masuoka et al. (2015) reported that the amount of β-glucan in electric pulse-treated samples was 1.22 times higher than in control samples.

Oh et al. (2009) developed a simple method for mushroom cultivation on a coniferous sawdust-based medium with molasses and wheat flour. They reported that cold shock for a day induced primordium formation. The sawdust medium of *Pseudotsuga menziesii* mixed with cottonseed meal, corn chip, wheat flour, and 10% molasses showed best productivity with 41%, followed by that of *Larix leptolepis* mixed with the same additives with 37% yield. Kurosumi et al*.* (2006) reported that mycelia raised by shaking liquid cultures shortens culturing time by about one third compared with conventional stationary liquid cultures. They suggested that this may be due to the more availability of dissolved oxygen and faster glucose consumption. At 30 g/L glucose concentration, pH 5 and temperature 25–30 °C, productivity of *S. crispa* mycelia reached its maximum.

Huang et al*.* (2007) used potato-dextrose-peptone agar (PDPA) medium for mycelial culture and pine sawdust with polished glutinous rice starch as a medium for basidiome production. The optimum temperature for mycelium growth and basidiome development was reported as 20–25 °C and 16–18 °C respectively, the optimum environment humidity as 90%-95%, and the optimum pH-value as 5–6. Yu et al*.* (2010) produced the mushroom using short logs of *Larix leptolepis, Pinus densiflora, P. rigida, *and *Quercus acutissima.* Mushroom production was highest on *P. rigida *logs. The optimal moisture content and temperature were 90 ~ 95% and 23–25 °C, respectively. Park et al*.* (2006) reported that the larch sawdust-based medium for the cultivation of *S. crispa *prepared as 0.76 g/cm^3^ in medium-density excluding particles less than 1 mm diam as best for mycelial growth. Jeong et al*.* (2011) reported that the optimal sugar content of media for mycelial growth was 6°Bx with 25 °C as the optimum temperature and a pH of 5.0.-6.0 for mycelial growth in liquid spawn, respectively. They used *Larix kaempferi* sawdust as the main material and observed that the mushroom yield, and cultivation period when supplemented with 20% corn flour and 10% wheat flour were highest and fastest, in terms of production respectively. Lu et al. (2011) investigated the effect of inorganic salts, vitamins, and phytohormones on the mycelial growth of *S. crispa*. Their result indicated that the growth rate of *S. crispa* reached its peak at concentrations of 1.0 g/L of magnesium sulfate, monopotassium phosphate, and sodium chloride. Among the vitamins, B1, B4 and B6 affected mycelial growth significantly; the best stimulatory vitamin for the growth was B4 at 8 mg/L. Among phytohormones, 6-BA and 6-KT stimulated the *S. crispa *growth.

Yanquan et al. (2012) carried out a study to determine the optimum technological parameters for the cultivation of *S. crispa*. Their results indicated that 22 °C was the optimum temperature for mycelial growth with 65% as the optimal moisture content, and amount of substrate material in the range of 300–315 g/bag satisfies the nutritional requirements for industrial cultivation. Yan Quan et al. (2012) examined the growth rates of *S. crispa* mycelium on potato dextrose agar (PDA) media and reported the maximum growth rate in the range 22–24 °C. For culturing the mushrooms, they used a substrate of 76% sawdust, 18% wheat bran, 2% cornmeal, 1.5% sucrose, 1.5% gypsum, and 1% calcium superphosphate. Mushroom yield was highest when the water content of the substrate was approximately 65% and the loading capacity was 900 g/bag. Ying et al. (2013) analysed the impact of exposure to light, varying in quality and quantity, on mycelial growth and primordium formation; they revealed that red light at 200 lx was most favourable for mycelial growth while yellow light was most conducive to primordium formation (Matsuura et al. 2020).

## CONCLUSION AND FUTURE PROSPECTS

*Sparassis crispa* is an important wild edible and medicinal mushroom now cultivated in many countries. Besides high protein and vitamin content, it also contains a variety of physiologically active compounds which exhibit anti-tumour, anti-microbial, anti-oxidant, anti-inflammatory, and anti-hypertensive activities. The immuno-modulating β-glucan from *S. crispa* plays a crucial role in modulating the immune response against cancer and has a stimulating effect on the innate immune system. It promotes wound healing in diabetic patients and shows anti-inflammatory activity via regulating the production of NO, implying that it could play a role in anti-inflammation therapy. The presence of phenolic compounds strengthens the antioxidant capacity. Extracts inhibit the growth of bacteria and fungi, indicating that the use of *S. crispa* in pharmaceuticals can protect humans from certain bacterial and fungal infections. The mushroom is considered edible all over the world, and no harmful effects have been recorded following human consumption. Polysaccharides of *S. crispa* also have potential for utilization in the food industries as a source of hydrocolloid (Choi et al. 2013). Therefore, using *S. crispa* as a dietary supplement may help in the treatment or be preventative of a variety of human diseases and ailments.

## Data Availability

Entire data has been added in the article itself.
